# Optimization of Polyphenol-Rich Extracts from Defatted Avocado Peel and Seed Residues Using Ultrasound-Assisted RSM: Antioxidant Potential and Valorization Prospects

**DOI:** 10.3390/antiox14111293

**Published:** 2025-10-28

**Authors:** Argyro Petrantonaki, Vasiliki-Ioanna Theodoropoulou, Martha Mantiniotou, Vassilis Athanasiadis, Eleni Bozinou, Stavros I. Lalas

**Affiliations:** Department of Food Science and Nutrition, University of Thessaly, Terma N. Temponera Street, 43100 Karditsa, Greece; apetrantonaki@uth.gr (A.P.); vtheodorop@uth.gr (V.-I.T.); mmantiniotou@uth.gr (M.M.); vaathanasiadis@uth.gr (V.A.); empozinou@uth.gr (E.B.)

**Keywords:** avocado by-products, peel oil, seed oil, fatty acid profile, response surface methodology, antioxidants, polyphenols, oxidative metrics, valorization, functional ingredients

## Abstract

It is well known that a significant amount of processed avocado fruit, specifically peel and seed, is treated as waste. During this study, these by-products were valorized through a two-step approach combining lipid profiling and optimized antioxidant extraction. Initially, oil was extracted and analyzed via GC-FID, revealing distinct fatty acid compositions: peel oil was rich in oleic (32.0%), linoleic (21.9%), and α-linolenic acids (9.7%), while seed oil was dominated by oleic (48.0%) and palmitic acids (20.7%), with moderate linoleic content (24.0%). Following defatting, the dried residues were subjected to ultrasound-assisted extraction, which was optimized by response surface methodology (RSM) to maximize the recovery of antioxidant-rich fractions. Peel extracts exhibited the highest total polyphenolic content (105.98 mg GAE/g), FRAP (673.89 μmol AAE/g), and ascorbic acid (17.9 mg/g), while seed extracts showed superior DPPH activity (1071.31 μmol AAE/g). Regression modeling identified optimal conditions for each antioxidant metric, highlighting matrix-specific bioactivity. The combined analysis of lipid and polar fractions underscores the multifunctional potential of avocado residues as sustainable sources of natural antioxidants. These findings support their application in food, cosmetic, and nutraceutical formulations, contributing to circular bioeconomy strategies.

## 1. Introduction

Human health depends on efficient metabolic processes, homeostatic balance, and properly functioning cellular repair mechanisms. Although free oxygen radicals play essential roles in normal physiology, their excess induces oxidative stress, leading to dysfunctions in the human body [1]. Reactive oxygen and nitrogen species are molecules characterized by the presence of at least one unpaired electron. This indicates that free radicals are extremely reactive molecules with brief lifespans that swiftly engage in chemical reactions with biological structures [2,3]. Free radicals are perpetually generated by every single cell under physiological conditions and impact our bodies daily [2]. To inhibit the interaction of radicals and biological molecules, antioxidants must be situated near the site of radical production, competing with the free radical for the biological substrate. Insufficient antioxidant levels to counteract reactive oxygen species (ROS) will lead to the deterioration of biomolecules, particularly lipid peroxidation, protein degradation (carbonylation of amino acid residues), oxidation of various DNA and RNA nucleotides, and enzyme inhibition, ultimately resulting in cellular apoptosis [4,5].

The investigation of naturally derived products for their antioxidant properties has garnered significant attention due to the demand for safer and more effective therapeutic alternatives, and the shift towards healthier lifestyles that incorporate natural antioxidant compounds [6]. Polyphenolic compounds, found in numerous plants and foods, have been thoroughly investigated for their antioxidant, antibacterial and anti-inflammatory properties [7,8]. Food by-products, often rich in polyphenolic compounds, are generated during plant-based food production but are usually discarded, contributing to environmental pollution [9]. In this context, scientists have concentrated on advancing sustainable food production, wherein the utilization of waste materials, referred to as byproducts, from agribusiness offers a significant chance for development [10,11].

In response to sustainable environmental practices, industry and scientists have been seeking to employ green extraction techniques on food residue and by-products in recent years [12]. Ultrasonication lies among the green extraction techniques, which is chiefly attributable to the prospective advantages of diminished extraction duration and less solvent waste [13,14]. The utilization of ultrasound for the targeted extraction of bioactive compounds facilitates a high yield at relatively low temperatures, hence limiting degradation and preserving the quality of the final extract [13]. Ultrasonication induces negative pressure in the liquid medium, resulting in acoustic cavitation, which appears as bubbles due to the inability of gases to remain dissolved under these conditions [15]. Ultrasound waves induce pressure fluctuations that produce cavitation bubbles, causing their development and subsequent collapse, which releases energy. The rupture of bubbles generates significant shear forces and localized elevated temperatures, resulting in cell disintegration and enhanced mass transfer, hence augmenting the yield of chemical compound extraction [16]. Some main factors that highly affect ultrasound efficiency are ultrasonic power, ultrasonic frequency and ultrasonic intensity [16].

Avocado, also known in the scientific community as *Persea americana* Mill., a member of the Lauraceae family, is a tropical fruit characterized by an olive green skin and a dense pale yellow flesh rich in oils [17]. Around 6 million tons of avocado fruit are harvested annually worldwide, generating 1.6 million tons of waste [18,19]. The elevated industrial processing and direct consumption are seen in its rapid and substantial production rise in 2020 and preceding years among tropical fruits, particularly for the widely favored ‘Hass’ variety [20]. Avocados generate a substantial amount of waste, including seeds, peels, and leaves, owing to both direct consumption and industrial processing. The avocado peel (AP) accounts for 11% and avocado seeds (ASs) account for 16% of the fruit’s total weight [21]. AP contains lipids (4.4–9.1%), carbohydrates (62–73.3%), fibers (~50%), minerals (1.5–6%), proteins (0.17–8%) and a high amount of polyphenolic compounds that result in a high antioxidant activity [22]. ASs are an excellent source of dietary fiber (2–4.2%) and contain lipids (3–15%), carbohydrates (43–81%), minerals (1.3–4.3%), proteins (0.14–9%) and rich bioactive compounds that can yield various useful substances, including phenolics, polyphenols, and tocopherols, which include antioxidant and antibacterial properties [18,23].

With all the above in mind, this study aims to examine the polyphenolic profile and antioxidant activity of avocado by-products. Until now, great importance has been given to the seed, and as a result, the peel and all the beneficial substances it contains have not been studied thoroughly. Research and applications underscore the potential of seed and peel extracts for integration into food systems, cosmeceuticals, and biodegradable packaging materials. Starch and polyphenols derived from seeds have been utilized to create biodegradable films possessing antioxidant and barrier properties, whereas peel extracts have exhibited protective effects against oxidative stress and apoptosis in neuronal cell models, owing to their high phenolic content [24]. In this study, both peel and seed will be examined simultaneously, with extraction parameters optimized using Response Surface Methodology (RSM). Ultrasound-assisted extraction (UAE) will be utilized as a green, non-thermal, beneficial extraction technique and parameters such as ultrasonic power, liquid-to-solid ratio, treatment duration, and appropriate solvent composition will be investigated. Ethanol, water and a 1:1 *v*/*v* mixture of the two will be utilized as solvents under investigation, due to terms of environmental protection and food-grade solvent usage. In addition, the oil composition in fatty acids of AS and AP will be studied, along with their possible antioxidant activity and oxidative stability.

## 2. Materials and Methods

### 2.1. Chemicals and Reagents

Deionized water was produced by a deionizing column. Dichloromethane and ethyl acetate were purchased from Carlo Erba (Normandie, France). The Human Corporation NEX Power 1000 device (Seoul, Republic of Korea) was utilized to produce ultrapure water. Sodium carbonate anhydrous (≥95.0% *w*/*w*), sodium chloride (≥99.0% *w*/*w*), chloroform, and ammonium thiocyanate (≥99.0% *w*/*w*) were purchased from Penta (Prague, Czech Republic). Ethanol (≥99.8% *v*/*v*), gallic acid (≥99.0% *w*/*w*), ammonium iron (II) sulfate hexahydrate (≥99.0% *w*/*w*), and Folin–Ciocalteu’s reagent were bought from Panreac Co. (Barcelona, Spain). Acetonitrile was purchased from Labkem (Barcelona, Spain). Trolox (6-hydroxy-2,5,7,8-tetramethylchroman-2-carboxylic acid) (≥96.5% *w*/*w*) was obtained from Glentham Life Sciences (Corsham, UK). Hydrogen peroxide (35%) was purchased from Chemco (Malsch, Germany). Iron (III) chloride hexahydrate (≥99.0% *w*/*w*) was from Merck (Darmstadt, Germany). L-ascorbic acid (≥99.0% *w/w*), trichloroacetic acid (≥99.0% *w*/*w*), aluminum chloride (≥99% *w*/*w*), hydrochloric acid (37% *w/w*), methanol (≥99.8% *v*/*v*), 2,2-diphenyl-1-picrylhydrazyl (DPPH) (≥90.0% *w*/*w*), 2,4,6-tris(2-pyridyl)-s-triazine (TPTZ) (≥98% *w*/*w*), FAME Mix C8–C24 reference standards, 2-propanol and polyphenolic chemical standards of HPLC grade (≥99.0% *w*/*w*) were all purchased from Sigma-Aldrich (Darmstadt, Germany).

### 2.2. Avocado Fruit Handling

**Figure 1:**
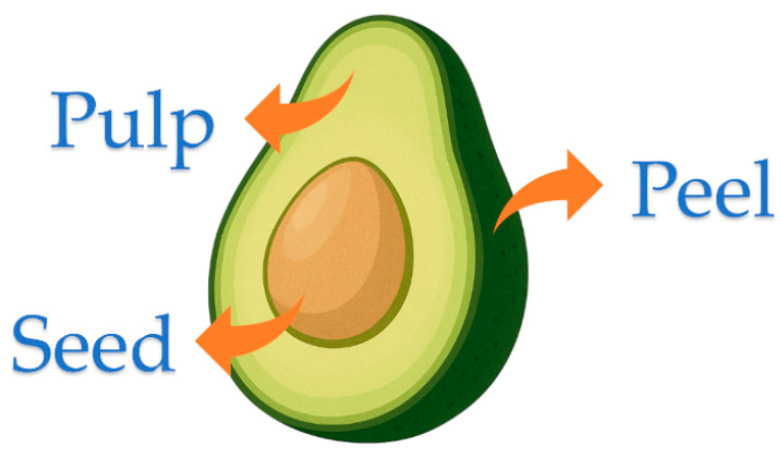
Anatomical parts of avocado fruit: pulp, peel, and seed.

Fresh avocado fruits (cv. Hass) were harvested by a local farmer in Agia village, Chania, Crete, Greece, coordinates: 35°28′19″ N 23°55′40″ E according to Google Earth (ver. 10.91.0.1 from Google Inc. (Cambridge, MA, USA). The field contains 180 avocado trees of the same cultivar within acres. Fruits were harvested on 3 March 2025 in unripe form in the afternoon so that they would not be damp. The harvesting was repeated in triplicate, and about 20 kg of fruit were collected each time. Unripe avocado fruits were transported to our lab and left at ambient temperature to ripen naturally. When the peel color turned from green to black, the fruits were ready to be consumed. The fruits were manually peeled, and the seeds were separated from the pulp and cracked to expose the inner kernel (Figure 1). Both peel and seed fractions were cut into small pieces, frozen at −40 °C, and subsequently freeze-dried using a BK-FD10P freeze-dryer (Biobase, Jinan, China), at −55 °C, 0.05 mbar until constant weight. The moisture content was measured as 74.33% for the AP and as 52.72% for AS. The dried materials were ground to a fine powder using a laboratory mill. The powder then underwent a sieving process with an Analysette 3 PRO apparatus from Fritsch GmbH (Oberstein, Germany), a total of five times for each sample. In both cases, particles with a diameter less than 400 μm were kept, with average particle diameters of 314 ± 60 μm for AP and 262 ± 11 μm for AS, and stored in airtight containers under vacuum at −40 °C until further use.

### 2.3. Defatting and Lipophilic Analysis

#### 2.3.1. Defatting Procedure

Freeze-dried avocado peel and seed powders were defatted with n-hexane at a ratio of 1:10 g/mL, under continuous mechanical stirring through a magnetic stirrer from Heidolph Instruments GmbH & Co. KG (Schwabach, Germany) at ambient temperature for 24 h. The solvent evaporated at decreased pressure with a rotary evaporator (40 °C), and the recovered oils were weighed to determine lipid yield. The defatted residues were air-dried to remove residual solvent and stored at −40 °C for subsequent polar extraction.

#### 2.3.2. Fatty Acid Profile

Fatty acid composition of the extracts was measured as previously established [25]. The recovered oils were esterified by base-catalyzed transesterification with methanolic KOH into fatty acid methyl esters (FAMEs). FAMEs were analyzed through a gas chromatograph coupled with a flame ionization detector (GC-FID) from Agilent Technologies (Santa Clara, CA, USA), Gas Chromatograph model 7890A, with a capillary column Omegawax (30 m × 320 μm × 0.25 μm) provided by Supelco (Bellefonte, PA, USA). Identification was conducted by the comparison of retention times with a certified FAME standard mixture (C8:0–C24:0). Results were presented as a relative proportion of the total detected fatty acids.

#### 2.3.3. Antioxidant Activity of Oils

The radical scavenging activity of the oils was examined via the DPPH assay, as established in an already published study [25]. Briefly, oil samples were dissolved in ethyl acetate, mixed with a DPPH solution (0.1 mM), and incubated in the dark for 30 min. Trolox was used as a positive control. Absorbance was measured at 515 nm utilizing a UV–1900i PharmaSpec double-beam spectrophotometer manufactured by Shimadzu (Kyoto, Japan), and results were expressed as μmol Trolox equivalents (TE)/g oil.

#### 2.3.4. Oxidative Stability Indices

Oxidative stability was assessed by measuring peroxide value (PV) according to the AOCS official method [26], expressed as mmol H_2_O_2_/kg oil, as previously discussed [25]. Specific extinction coefficients at 232 nm (K232) and 270 nm (K270) were determined spectrophotometrically in cyclohexane, providing information on primary and secondary oxidation products, respectively, as previously discussed [25].

#### 2.3.5. Lipophilic Pigments

Spectrophotometric analysis of lipophilic pigments was performed on avocado peel and seed oil extracts dissolved in diethyl ether at a concentration of 0.3 g oil/mL. Measurements were carried out in 1.00 cm quartz cuvettes using a UV–Vis spectrophotometer (400–800 nm). For the peel extract, a 10-fold dilution was applied to avoid detector saturation due to high pigment content.

Carotenoid absorption maxima were identified in the 400–500 nm region, and chlorophylls in the 640–680 nm region. The %III/II ratio, defined as the ratio of the third to the second carotenoid absorption peak, was calculated to assess spectral fine structure and support pigment identification [27]:

(1) $$\%\mathrm{III}/\mathrm{II}=\left(\frac{A_{\mathrm{III}}}{A_{\mathrm{II}}}\right)\;\times \;100$$

where *A*_II_ and *A*_III_ are the absorbance values of the second and third carotenoid peaks, respectively.

Quantification of carotenoids was performed using the Beer-Lambert law:

(2) $$C_{\mathrm{carotenoid}\;}(\mu g/\mathrm{mL})=\frac{A_{\lambda }\cdot V\cdot 10^{4}}{A^{1\%}}$$

where *A*_λ_ is the absorbance at λ_max_, *V* is the dilution factor, and *A*^1%^ is the specific absorption coefficient for the assigned carotenoid (2500 for carotenoids in diethyl ether) [27]. Final concentrations were expressed in mg pigment/kg oil using the known oil density:

(3) $$C_{\mathrm{oil}\;}(\mathrm{mg}/\mathrm{kg})=\frac{C\;(\mu g/\mathrm{mL})}{0.3}$$

Chlorophyll a was quantified in the diluted peel oil extract using the Wellburn and Lichtenthaler [28] equation adapted for diethyl ether:

(4) $$C_{a}\;(\mu g/\mathrm{mL})=(16.36\times A_{663.2}\times F_{D})-(2.43\times A_{646.8}\times F_{D})$$

where *F*_D_ denotes the dilution factor. The resulting concentration was corrected for the 10-fold dilution and converted to mg/kg oil as described above.

### 2.4. Experimental Design

A custom RSM design was employed to optimize the ultrasound-assisted extraction (UAE) of polar bioactive compounds from defatted avocado peel and seed residues. Five independent variables were considered: waste type (categorical: peel or seed), ultrasonic power (*E*), liquid-to-solid ratio (*R*), ethanol concentration (*C*), and extraction time (*t*). Continuous factors were studied at three coded levels (−1, 0, +1), corresponding to the actual values shown in Table 1. The ultrasonication (US) process was executed through an Elmasonic P70H bath obtained from Elma Schmidbauer GmbH (Singen, Germany). An EWJ 600–2M precision scale from Kern (Frankfurt, Germany) was employed to weigh the freeze-dried and ground material, while liquid supernatant separation from the solid byproduct was conducted through a NEYA 16R centrifuge from Remi Elektrotechnik Ltd. (Palghar, India), spinning the samples at 4500 rpm for 5 min. A total of 26 randomized experimental runs were performed. The responses evaluated were TPC, FRAP, DPPH radical scavenging activity, and AAC. Regression models were fitted to the data, and analysis of variance (ANOVA) was used to assess model significance [29]. Optimal conditions were determined using desirability functions to maximize antioxidant responses.

In addition to the Custom design, a stepwise multiple regression analysis was performed to identify the most significant linear, interaction, and quadratic terms affecting the extraction responses. The final predictive model for each response was expressed as a second-order polynomial equation of the form:

(5) $$Y_{k}=\beta_{0}+\sum_{i=1}^{2}\beta_{i}X_{i}+\sum_{i=1}^{2}\beta_{\mathrm{ii}}X_{i}^{2}+\sum_{i=1}^{2}\sum_{j=i+1}^{3}\beta_{\mathrm{ij}}X_{i}X_{j}$$

where the independent variables are denoted by *X_i_* and *X_j_*, and the predicted response variable is defined by *Y_k_*. In the model, the intercept and regression coefficients *β*_0_, *β_i_*, *β_ii_*, and *β_ij_* represent the linear, quadratic, and interaction terms, respectively.

### 2.5. Extraction of Polar Compounds from Defatted Residues

Defatted powders of peel and seed were subjected to UAE under the conditions specified by the experimental design. Extractions were carried out in an ultrasonic bath with controlled temperature (<30 °C) to avoid degradation of thermolabile compounds. Ethanol–water mixtures were used as solvents according to the design matrix. After extraction, the mixtures were centrifuged at 4500 rpm for 10 min, filtered, and the supernatants were stored at −40 °C until analysis.

### 2.6. Determination of Antioxidant Parameters

#### 2.6.1. Measurement of Total Polyphenolic Content (TPC)

A previous spectrophotometric approach [30] was used to quantify. Using a calibration curve for gallic acid, it was feasible to determine the TPC. Briefly, 200 μL of the sample was mixed with 200 μL of Folin–Ciocalteu reagent, and after 2 min, 1600 μL of 5% *w*/*v* aqueous sodium carbonate solution was added in a 2-mL Eppendorf tube. The mixture was incubated at 40 °C for 20 min and the absorbance was recorded at 740 nm. TPC was calculated in milligrams of gallic acid equivalents (GAE) for every gram of dry weight (dw), according to Equation (6), using a gallic acid calibration curve (R^2^ = 0.9995) ranging from 0 to 100 mg/L.

(6) $$\mathrm{TPC}\;(\mathrm{mg}\;\mathrm{GAE}/g\;\mathrm{dw})=\frac{C_{TP}\times V}{w}$$

where the volume of the extraction medium is indicated with *V* (expressed in L) and the dry weight of the sample as *w* (expressed in g).

#### 2.6.2. Measurement of Ferric Reducing Antioxidant Power (FRAP)

In order to assess FRAP, a well-established method [31] was employed. In a 2-mL Eppendorf tube, 100 μL of properly diluted sample was mixed with 100 μL of FeCl_3_ solution (4 mM in 0.05 M HCl). The mixture was incubated at 37 °C for 30 min, with 1800 μL of TPTZ solution (1 mM in 0.05 M HCl) immediately added right after, and the absorbance was measured after 5 min at 620 nm. The absorbance was measured at 620 nm. A calibration curve (R^2^ = 0.9953) for ascorbic acid in 0.05 M HCl with values ranging from 50 to 500 μM was used to determine the ferric-reducing power (*P*_R_). *P*_R_ was determined as μmol of ascorbic acid equivalents (AAE) per g of dw, as shown in Equation (7).

(7) $$P_{R}\;(\mu \mathrm{mol}\;\mathrm{AAE}/g\;dw)=\frac{C_{AA}\times V}{w}$$

where the volume of the extraction medium is indicated with *V* (expressed in L) and the dry weight of the sample as *w* (expressed in g).

#### 2.6.3. Measurement of DPPH Radical Scavenging Activity

Using an already established DPPH^•^ technique [30], the antioxidant activity of the extracts was further evaluated by assessing their ability to scavenge the DPPH radical. In brief, 50 μL of the sample was mixed with a quantity of 1950 μL of a 100 μM DPPH^•^ solution in methanol, with the solution being kept at room temperature for 30 min in the dark. Then, at 515 nm, the absorbance was recorded. In addition, the absorbance was tested instantly using a blank sample in place of the solution containing DPPH^•^ and methanol. The inhibition % was determined and the results were given as μmol AAE per g of dw (Equation (8)) utilizing an ascorbic acid calibration curve in methanol (0–1000 μM, R^2^ = 0.9926, Equation (9)).

(8) $$\%\;\mathrm{Scavenging}=\frac{A_{\mathrm{control}}-A_{\mathrm{sample}}}{A_{\mathrm{control}}}\times 100$$

(9) $$A_{\mathrm{AR}}\;(\mu \mathrm{mol}\;\mathrm{AAE}/g\;dw)=\frac{C_{AA}\times V}{w}$$

where the volume of the extraction medium is indicated with *V* (expressed in L) and the dry weight of the sample as *w* (expressed in g).

#### 2.6.4. Measurement of Ascorbic Acid Content

AAC was evaluated through a method successfully used in the past [32]. Briefly, 100 μL of the sample was mixed with 500 μL of 10% *v*/*v* Folin–Ciocalteu’s reagent (in water) and 900 μL of 10% *w*/*v* TCA. After 10 min, the absorbance was recorded at 760 nm. A calibration curve of ascorbic acid (R^2^ = 0.9980) with concentrations ranging from 0 to 500 mg/L was utilized to assess the results (Equation (10)).

(10) $$\mathrm{AAC}\;(\mathrm{mg}\;/g\;\mathrm{dw})=\frac{C_{AA}\times V}{w}$$

where the volume of the extraction medium is indicated with *V* (expressed in L) and the dry weight of the sample as *w* (expressed in g).

#### 2.6.5. HPLC Analysis

**Figure A1:**
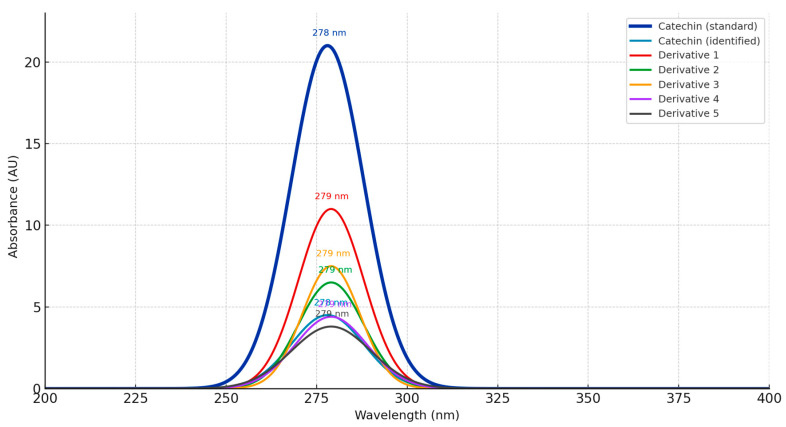
Overlay of UV–Vis spectra (200–400 nm) for catechin standard and the five identified catechin derivatives.

An HPLC system by Shimadzu Europa GmbH (Duisburg, Germany) was utilized to identify and quantify individual polyphenols of the extracts. A CBM–20A HPLC model was coupled to an SPD-M20A diode array detector (DAD). The separation of the compounds was feasible through a Luna C18(2) column (100 Å, 5 μm, 4.6 mm × 250 mm) by Phenomenex Inc. (Torrance, CA, USA). The HPLC analysis of specific polyphenols from the extracts originated from our previous research [32]. The mobile phase comprised 0.5% formic acid in acetonitrile (B) and 0.5% formic acid in an aqueous solution (A). The gradient program commenced at 0%, escalating to 40% B, then reaching 50% B over 10 min, followed by 70% B in an additional 10 min, and maintaining a constant value for 10 min. The flow rate of the mobile phase was maintained at a constant 1 mL/min. The compounds were identified and quantified through evaluating their absorbance spectra and retention times to those of pure standards, using calibration curves (0–50 μg/mL). In Table A1, the equations, along with R^2^, retention times, UV_max_, LOD, and LOQ of all identified compounds, are provided. To support the classification of catechin derivatives, Figure A1 presents an overlay of UV-Vis spectra (200–400 nm) comparing the catechin standard with the five identified compounds.

### 2.7. Color Analysis

The Lovibond CAM-System 500 colorimeter by The Tintometer Ltd. (Amesbury, UK) was used to evaluate the CIELAB factors (L*, a*, and b*) of the samples. The color of the extracts following an already published protocol [32]. The L* coordinate defines brightness, ranging from 0 (black) to 100 (white), and the a* axis signifies the red–green dimension, with positive values indicating red and negative ones indicating green. Colors can be assessed by their b* values, which reflect their inclination towards yellow (positive values) or blue (negative values), respectively.

### 2.8. Statistical Analysis

Every statistical process conducted was through the JMP^®^ Pro 16.0.0 (SAS Institute Inc., Cary, NC, USA). Mean ± standard deviation (SD) of at least three independent determinations (*n* = 3) was used as the final data presentation. The Shapiro–Wilk test was implemented to evaluate the normality of the data before its analysis. Comparisons between avocado peel and seed oils were conducted using one-way analysis of variance (ANOVA) followed by Tukey–Kramer’s HSD post hoc test to identify significant variations among means. Differences were considered statistically significant at *p* < 0.05. Multivariate analyses (principal component analysis, PCA, and partial least squares regression, PLS) were applied exclusively during the optimization of the extraction process, in order to evaluate the influence of experimental factors, identify significant variables, and guide the selection of optimal extraction conditions.

## 3. Results and Discussion

### 3.1. Peel and Seed Oils Analysis

#### 3.1.1. Fatty Acid Composition

The fatty acid profiles of avocado peel and seed oils revealed distinct lipid signatures (Table 2). Both oils were rich in oleic acid (C18:1), with seed oil showing a higher content (48.09 ± 2.31%) compared to peel oil (42.05 ± 3.11%), although the difference was not statistically significant (*p* > 0.05). Saturated fatty acids (SFA) were also more abundant in seed oil (26.80 ± 0.69%) than in peel oil (24.28 ± 1.06%, *p* < 0.05), mainly due to higher levels of stearic acid (C18:0, 4.70%) and, and to a lesser extent, lignoceric acid (C24:0, 0.94%), while palmitic acid (C16:0) was present at comparable levels in both oils (~20%). Peel oil, on the other hand, was significantly richer in polyunsaturated fatty acids (PUFA), especially α-linolenic acid (C18:3, ω-3), which reached 9.72 ± 0.70% compared to only 0.61 ± 0.02% in seed oil (*p* < 0.001). This resulted in a markedly lower ω-6/ω-3 ratio in peel oil (2.25) versus seed oil (39.43), indicating superior anti-inflammatory potential [25,33]. Minor fatty acids such as lauric (C12:0), myristic (C14:0), behenic (C22:0), and erucic (C22:1) were detected in trace amounts, with lauric acid being notably higher in peel oil (3.17 ± 0.19%) than in seed oil (0.17 ± 0.01%). The PUFA:SFA ratio was significantly higher in peel oil (1.30 ± 0.04) compared to seed oil (0.92 ± 0.01), while the MUFA:PUFA ratio was higher in seed oil (1.95 ± 0.02) than in peel oil (1.39 ± 0.00), reflecting the dominance of oleic acid in the seed fraction.

#### 3.1.2. Oxidative Stability and Antioxidant Activity

The oxidative stability and antioxidant activity of avocado peel and seed oils were evaluated through DPPH radical scavenging activity, conjugated dienes (CDs), conjugated trienes (CTs), and peroxide value (PV) (Table 3). Both oils exhibited comparable DPPH activity, with peel oil showing slightly higher values (15.49 ± 0.91 mmol TEAC/kg) than seed oil (14.97 ± 0.66 mmol TEAC/kg), although the differentiation was not of statistical significance (*p* > 0.05). However, significant differences were observed in oxidative degradation markers. Peel oil had higher peroxide values (PV) (16.95 ± 0.76 mmol H_2_O_2_/kg) compared to seed oil (13.50 ± 0.27 mmol H_2_O_2_/kg, *p* < 0.05), indicating greater primary oxidation. Similarly, CDs and CTs were markedly elevated in peel oil (33.56 ± 2.52 and 10.52 ± 0.36 mmol/kg, respectively) versus seed oil (11.51 ± 0.51 and 1.67 ± 0.13 mmol/kg, *p* < 0.001), reflecting enhanced formation of secondary oxidation products. These results are consistent with the fatty acid profiles, where peel oil contained significantly higher levels of PUFA (especially C18:3), rendering it more susceptible to oxidative degradation. Despite this, its antioxidant activity remained slightly superior, likely due to the presence of tocopherols and polyphenolic compounds co-extracted with the oil.

#### 3.1.3. Pigment Composition and Spectral Characteristics

**Figure 2:**
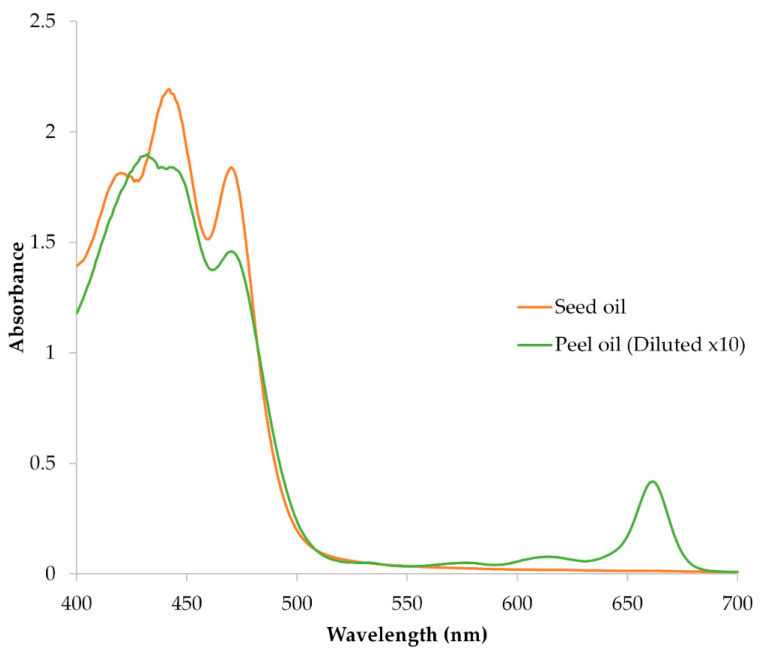
UV–Vis absorbance spectra of avocado peel and seed oil extracts in diethyl ether. Seed oil shows three carotenoid maxima typical of lutein-type xanthophylls, while peel oil exhibits overlapping carotenoid and chlorophyll a peaks.

UV–Vis spectral analysis revealed distinct pigment profiles in avocado peel and seed oils (Figure 2). The seed oil exhibited three carotenoid absorption maxima at 420, 442, and 470 nm, with a %III/II ratio of 83.8%, consistent with lutein-type xanthophylls. The peel oil showed two carotenoid peaks at 432 and 470 nm, alongside a strong chlorophyll a absorption at 661 nm, indicating a mixed pigment composition. Quantitative estimates based on specific extinction coefficients suggested that peel oil contained 253.1 ± 15.95 mg/kg of α-carotene-equivalent and 209.4 ± 9.76 mg/kg of chlorophyll a, while seed oil contained 29.2 ± 2.16 mg/kg of lutein-equivalent (Table 4). These results confirm the pigment-rich nature of peel oil, with nearly 9-fold higher carotenoid content and substantial chlorophyll a levels compared to seed oil. While this enhances its antioxidant potential, it also increases susceptibility to photooxidative degradation, especially under light exposure [34]. The pigment concentrations reported in Table 4 are consistent with literature values for carotenoid- and chlorophyll-rich fruit residues, and reflect the high extraction efficiency and spectral clarity achieved using diethyl ether as solvent [35,36].

Taken together, the fatty acid profiles, oxidative stability markers, and pigment composition reveal complementary properties of avocado peel and seed oils. Seed oil offers greater oxidative resistance and a safer pigment profile dominated by lutein, while peel oil provides a richer source of carotenoids and chlorophylls, with enhanced antioxidant potential but increased susceptibility to degradation. These findings support the targeted valorization of each fraction for distinct functional applications, provided appropriate stabilization and formulation strategies are employed.

### 3.2. Experimental Results of the RSM Design

The experimental results of the 26 design points are presented in Table 5. TPC values ranged from 3.72 to 103.14 mg GAE/g dw, FRAP from 61.61 to 602.39 µmol AAE/g dw, DPPH from 53.58 to 1103.76 µmol AAE/g dw, and AAC from 1.90 to 17.19 mg/g dw. The highest TPC was observed in peel extracts at 100% ultrasonic power and 50% ethanol (Run 17), while the strongest DPPH activity was obtained in seed extracts at 100% ultrasonic power and 100% ethanol (Run 10). These results highlight the strong dependence of antioxidant responses on both matrix type and extraction conditions.

The regression analysis revealed distinct but complementary patterns across the four antioxidant responses (Table 6). TPC and FRAP were consistently higher in peel extracts, with ethanol concentration emerging as the most influential factor. Both responses showed strong quadratic effects of ethanol (optimum ≈ 50% *v*/*v*), confirming that intermediate solvent polarity maximizes polyphenolic recovery and reducing power. FRAP was additionally enhanced by higher liquid-to-solid ratios, indicating that solvent availability plays a critical role in extracting reducing compounds. In contrast, DPPH activity was markedly stronger in seed extracts, highlighting compositional differences between the two matrices. DPPH was positively influenced by ethanol concentration, solvent ratio, and ultrasonic power, with several significant two-factor interactions (e.g., Ratio × Ethanol, Waste × Ethanol, Power × Ratio). These results suggest that radical scavenging compounds in seeds are more efficiently released under harsher extraction conditions. For AAC, peel extracts consistently yielded higher values, with solvent ratio being the dominant positive factor. Ethanol concentration exerted a negative linear effect, again with a quadratic optimum at intermediate levels. Strong interactions (Waste × Ratio, Waste × Ethanol) confirmed that peel matrices respond differently to solvent composition compared to seeds. Overall, the models demonstrated high predictive power (R^2^ > 0.93 for all responses, up to 0.989 for AAC). The consistent significance of ethanol concentration and its quadratic term across all responses underscores the importance of solvent polarity in balancing the extraction of both hydrophilic and moderately lipophilic antioxidants. The divergence between peel (higher TPC, FRAP, AAC) and seed (higher DPPH) highlights the complementary bioactive potential of the two by-products, supporting their combined valorization [37].

### 3.3. Model Analysis

The experimental data obtained from the custom RSM design were fitted to second-order polynomial models. Non-significant terms (*p* > 0.05) were removed through stepwise regression, resulting in reduced quadratic equations with high coefficients of determination (R^2^ = 0.93–0.99). To preserve model hierarchy, linear terms were retained when their corresponding quadratic or interaction terms were significant. The final reduced models are presented below (Equations (11)–(14)), containing only statistically significant predictors (*p* < 0.05).

The fitted models for TPC, FRAP, DPPH radical scavenging activity, and AAC are presented below in coded variables. Waste type was included as a categorical factor (*W* = +1 for peel, *W* = −1 for seed):

(11) *TPC* = 98.5 − 1.43*X*_2_ + 1.84*X*_4_ + 0.0105*X*_2_^2^ − 0.0190*X*_4_^2^ + 0.125*X*_5_^2^ + 0.0049*X*_2_*X*_3_ − 0.0025*X*_2_*X*_4_ + 0.0026*X*_3_*X*_4_ + *W*(12.6 + 0.120*X*_2_ − 0.286*X*_4_)

(12) *FRAP* = 94.9 − 10.2*X*_2_ + 11.2*X*_3_ + 5.85*X*_4_ + 23.8*X*_5_ + 0.094*X*_2_^2^ − 0.065*X*_4_^2^ − 0.042*X*_2_*X*_3_ − 0.135*X*_2_*X*_5_ − 0.034*X*_3_*X*_4_ − 0.271*X*_3_*X*_5_ + *W*(44.7 − 55.6 − 1.59*X*_4_)

(13) *DPPH* = 259.1 − 2.32*X*_2_ + 3.58*X*_3_ − 1.80*X*_4_ − 11.7*X*_5_ + 0.700*X*_5_^2^ + 0.0586*X*_2_*X*_3_ + 0.0326*X*_2_*X*_4_ + 0.0618*X*_3_*X*_4_ − 0.208*X*_3_*X*_5_ − 0.0874*X*_4_*X*_5_ + *W*(259.0 − 3.03*X*_2_ + 0.637*X*_3_ − 1.72*X*_4_ + 3.93*X*_5_)

(14) *AAC* = 1.85 + 0.00431*X*_2_ + 0.168*X*_3_ − 0.00519*X*_4_ + 0.139*X*_5_ − 0.000292*X*_4_^2^ + 0.000256*X*_2_*X*_4_ − 0.00167*X*_2_*X*_5_ + *W*(1.09 + 0.0511*X*_3_ − 0.0299*X*_4_)

**Figure 3:**
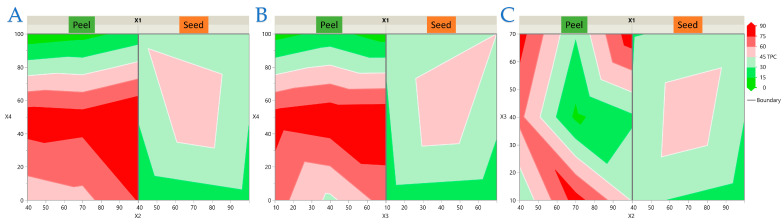
Response surface and contour plots for total polyphenolic content (TPC) of avocado peel and seed extracts. Panels illustrate the combined effects of (**A**) Power (*X*_2_) × Ethanol concentration (*X*_4_), (**B**) Ratio (*X*_3_) × Ethanol concentration (*X*_4_), and (**C**) Power (*X*_2_) × Ratio (*X*_3_). Sample type (*X*_1_: peel or seed) is shown separately in each panel. All other factors (*X*_5_) were fixed at their central levels. Warmer colors indicate higher predicted values.

**Figure 4:**
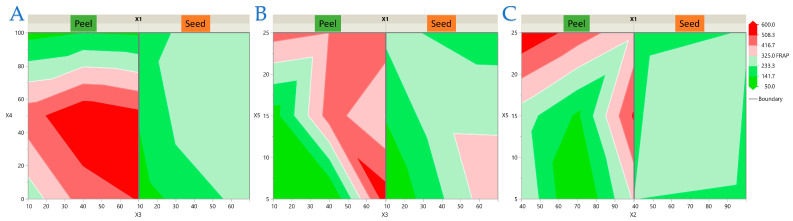
Response surface and contour plots for ferric reducing antioxidant power (FRAP) of avocado peel and seed extracts. Panels illustrate the combined effects of (**A**) Ratio (*X*_3_) × Ethanol concentration (*X*_4_), (**B**) Ratio (*X*_3_) × Time (*X*_5_), and (**C**) Power (*X*_2_) × Time (*X*_5_). Sample type (*X*_1_: peel or seed) is shown separately in each panel. All other factors were fixed at their central levels. Warmer colors indicate higher predicted values.

**Figure 5:**
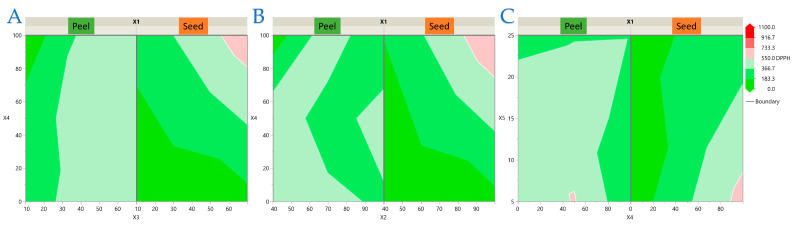
Response surface and contour plots for DPPH radical scavenging activity of avocado peel and seed extracts. Panels illustrate the combined effects of (**A**) Ratio (*X*_3_) × Ethanol concentration (*X*_4_), (**B**) Power (*X*_2_) × Ethanol concentration (*X*_4_), and (**C**) Ethanol concentration (*X*_4_) × Time (*X*_5_). Sample type (*X*_1_: peel or seed) is shown separately in each panel. All other factors were fixed at their central levels. Warmer colors indicate higher predicted values.

**Figure 6:**
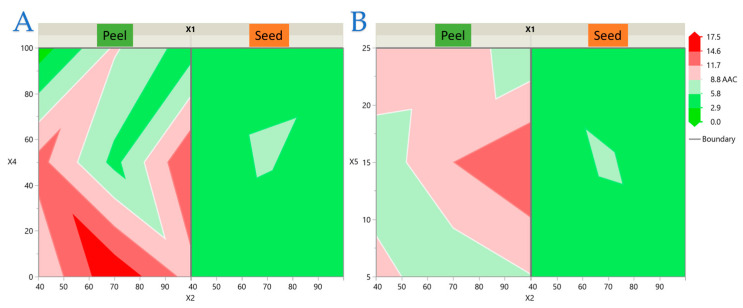
Response surface and contour plots for ascorbic acid content (AAC) of avocado peel and seed extracts. Panels illustrate the combined effects of (**A**) Power (*X*_2_) × Ethanol concentration (*X*_4_), and (**B**) Power (*X*_2_) × Time (*X*_5_). Sample type (*X*_1_: peel or seed) is shown separately in each panel. All other factors were fixed at their central levels. Warmer colors indicate higher predicted values.

The interaction plots provide additional insight into the regression models by visually confirming the combined effects of the experimental factors on each antioxidant response. These graphical representations complement the statistical outputs and facilitate the interpretation of matrix-dependent differences between peel and seed extracts. To visualize the interaction effects identified in the regression models, response surface and contour plots were generated for each antioxidant response of avocado peel and seed extracts. For clarity, the results are presented in four separate figures: Figure 3 (TPC), Figure 4 (FRAP), Figure 5 (DPPH), and Figure 6 (AAC). Each figure contains three (or two for AAC) representative two-factor interactions, with the sample type (*X*_1_: peel or seed) shown separately. All other factors were fixed at their central levels. This arrangement highlights the most influential parameter combinations and allows direct comparison of matrix-dependent trends. As shown in Figure 3, Figure 4, Figure 5 and Figure 6, peel extracts generally exhibited higher TPC, FRAP, and AAC values, whereas seed extracts aligned more strongly with DPPH activity. These visualizations confirm the statistical findings and provide an intuitive representation of how factor interactions drive antioxidant capacity in each matrix.

The optimization profiler identified distinct extraction conditions for each antioxidant response (Table 7). TPC was maximized in peel extracts (105.98 ± 12.94 mg GAE/g) under high power, moderate ratio, and low ethanol concentration. FRAP also peaked in peel extracts (673.89 ± 141 µmol AAE/g) at a low ratio and intermediate ethanol. In contrast, DPPH activity was highest in seed extracts (1071.31 ± 131.16 µmol AAE/g) under harsher conditions (high ratio, low ethanol, high power). Finally, AAC reached its maximum in peel extracts (17.9 ± 1.18 mg/g) at a low ratio and extended extraction time. All models achieved high desirability values (>0.87), confirming the robustness of the optimization. These findings highlight the complementary bioactive potential of avocado peel (rich in polyphenols, reducing power, and ascorbic acid) and seed (strong radical scavenging activity).

### 3.4. Impact of Extraction Parameters on Antioxidant Assays Through Pareto Plot Analysis

**Figure 7:**
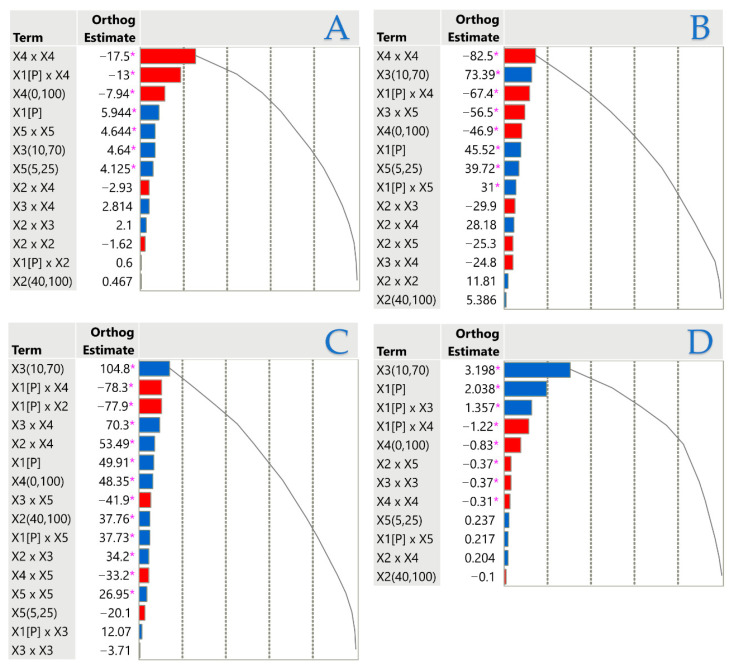
Normalized Pareto plots showing the relative impact of extraction parameters on (**A**) TPC, (**B**) FRAP, (**C**) DPPH, and (**D**) AAC of avocado peel and seed extracts. Blue bars denote positive effects; red bars denote negative effects. The dashed line represents the *t*-ratio threshold for statistical significance (*p* < 0.05), with significant factors marked by asterisks.

The relative influence of the extraction parameters on each antioxidant response was further evaluated through Pareto plot analysis (Figure 7). The standardized effects highlight both the magnitude and direction of each factor, with statistical significance determined at *p* < 0.05. For TPC, ethanol concentration (*X*_4_) was the dominant factor, with a strong negative linear effect and a highly significant quadratic term (*X*_4_^2^), indicating an optimum at low–moderate ethanol levels. Waste type (*X*_1_) was also significant, with peel extracts yielding higher TPC than seeds. Additional curvature effects of power (*X*_2_^2^) and time (*X*_5_^2^) suggested optima at intermediate levels, while solvent-to-solid ratio (*X*_3_) had a negligible impact. In the case of FRAP, solvent ratio (*X*_3_) exerted the strongest positive effect, while ethanol concentration (*X*_4_) again showed a negative linear and quadratic influence. Waste type (*X*_1_) favored peel extracts, and significant interactions (*X*_1_ × *X*_4_, *X*_3_ × *X*_5_) revealed that ethanol and time modulated the efficiency of polyphenolic recovery differently across matrices. Power contributed mainly through a quadratic effect (*X*_2_^2^), suggesting an optimum at intermediate intensity. The DPPH model was the most interaction-rich. Ratio (*X*_3_), ethanol (*X*_4_), and power (*X*_2_) were strong positive drivers, whereas time (*X*_5_) had a negative effect. Numerous interactions were significant, including Waste × Power (*X*_1_ × *X*_2_), Waste × Ethanol (*X*_1_ × *X*_4_), Ratio × Ethanol (*X*_3_ × *X*_4_), and Ratio × Time (*X*_3_ × *X*_5_). These results confirm that seed extracts, under conditions of high ratio, high ethanol, and strong ultrasonic power, achieved the highest radical scavenging activity, while prolonged extraction reduced activity. For AAC, solvent ratio (*X*_3_) and waste type (*X*_1_) were the most influential positive factors, with peel extracts showing higher ascorbic acid content. Ethanol concentration (*X*_4_) had a strong negative effect, both linear and quadratic, indicating that moderate ethanol levels are optimal. Significant interactions (*X*_1_ × *X*_3_, *X*_1_ × *X*_4_) highlighted the matrix-dependent response, with peel extracts benefiting more from a high ratio but being more sensitive to ethanol increase. Additional interactions (*X*_2_ × *X*_4_, *X*_2_ × *X*_5_) suggested that power and time jointly modulate ascorbic acid stability. Overall, Pareto analysis confirmed that ethanol concentration and waste type were the most consistent determinants across assays, while ratio, power, and time exerted assay-specific effects. Peel extracts were superior in TPC, FRAP, and AAC, whereas seed extracts excelled in DPPH. These findings align with the optimization results and highlight the complementary bioactive potential of avocado peel and seed.

### 3.5. Principal Component Analysis (PCA) and Multivariate Component Analysis (MCA)

**Figure 8:**
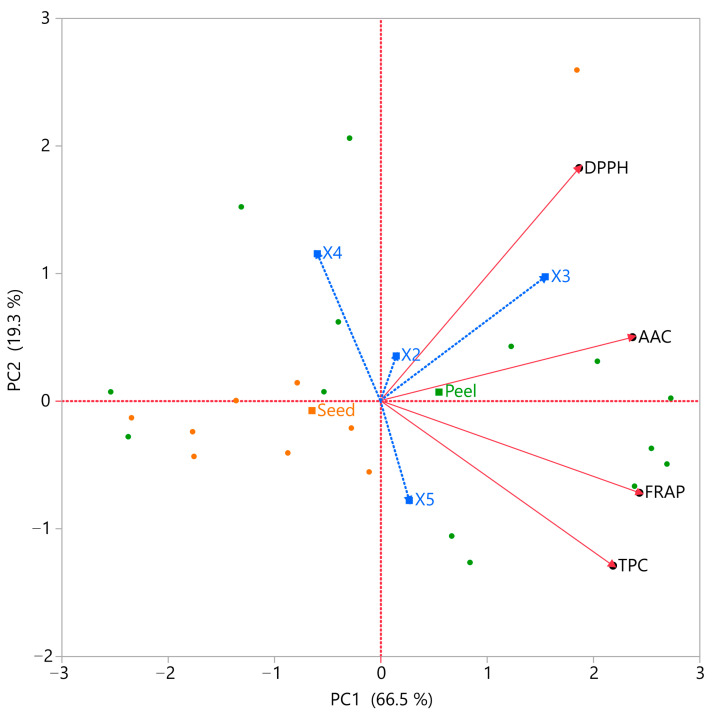
Principal Component Analysis (PCA) biplot of avocado peel and seed extracts based on TPC, FRAP, DPPH, and AAC responses. PC1 is mainly associated with TPC, FRAP, and AAC, while PC2 is driven by DPPH. Peel extracts cluster toward higher TPC, FRAP, and AAC, whereas seed extracts align with DPPH. Design points are color-coded to distinguish peel and seed samples.

PCA and MCA were applied to explore the interrelationships among the antioxidant ability of AP and AS extracts (Figure 8, Table 8). The correlation matrix revealed strong positive interactions between TPC and FRAP (r = 0.74), as well as between FRAP and AAC (r = 0.69), confirming that polyphenolic compounds and ascorbic acid jointly contribute to reducing power. Moderate correlations were observed between AAC and DPPH (r = 0.59) and between TPC and AAC (r = 0.53), while DPPH showed only weak correlation with TPC (r = 0.31) and moderate correlation with FRAP (r = 0.43). Variable clustering grouped all four assays into a single cluster, with FRAP identified as the most representative variable, explaining 66.5% of the total variation. Within this cluster, FRAP and AAC exhibited the highest R^2^ with the cluster (0.80 and 0.75, respectively), followed by TPC (0.64) and DPPH (0.47). This indicates that while all assays are interrelated, FRAP best captures the shared variance across the dataset. The PCA biplot further supported these findings, with TPC, FRAP, and AAC loading strongly on the first principal component (PC1), while DPPH contributed more distinctly to PC2, separating seed extracts from peel extracts. This multivariate structure highlights the complementary nature of the assays: peel extracts are characterized by higher polyphenols, reducing power, and ascorbic acid, whereas seed extracts are distinguished by stronger radical scavenging activity. Collectively, these multivariate analyses demonstrate that while peel extracts are enriched in polyphenols and ascorbic acid, seed extracts excel in radical scavenging. This complementary behavior underscores the importance of integrating multiple assays and multivariate tools (PCA, MCA) to capture the full spectrum of antioxidant potential in avocado by-products.

### 3.6. Partial Least Squares (PLS) Analysis

**Figure 9:**
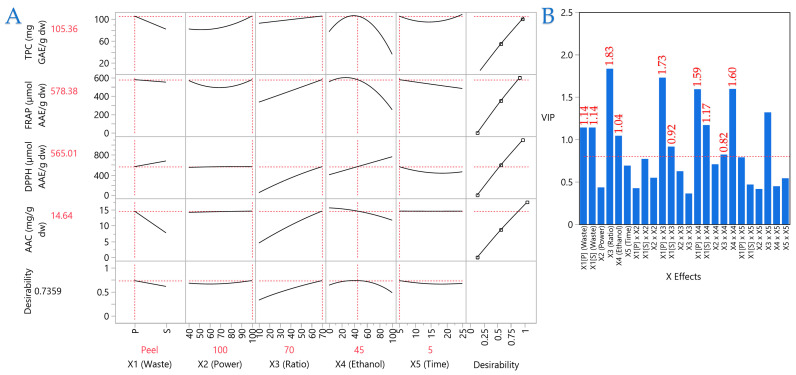
Partial Least Squares (PLS) regression results for avocado peel and seed extracts. (**A**) Prediction Profiler illustrating the effect of extraction parameters on TPC, FRAP, DPPH, and AAC. (**B**) Variable Importance in Projection (VIP) scores identifying the most influential extraction parameters. A red dashed line marks the 0.8 significance threshold, indicating the relative importance of each variable in the model.

PLS regression was employed to optimize the extraction conditions and validate the predictive performance of the model. The PLS model with 15 latent factors explained 85.8% of the total variance in *X* and 99.98% of the variance in *Y*, with cross-validation indicating that the first 7–8 factors contributed most to predictive ability. The Prediction Profiler (Figure 9A) illustrated the response surfaces for each assay, confirming that moderate ethanol concentration, optimized solvent ratio, and peel matrix favored higher TPC, FRAP, and AAC, whereas seed extracts were associated with enhanced DPPH activity. The Variable Importance in Projection (VIP) scores (Figure 9B) identified the solvent-to-solid ratio (*X*_3_), the interactions of waste type with ratio (*X*_1_ × *X*_3_) and ethanol (*X*_1_ × *X*_4_), and the quadratic effect of ethanol (*X*_4_^2^) as the most influential factors (VIP > 1.5).

The optimized conditions (*X*_1_ = peel, *X*_2_ = 100 W, *X*_3_ = 70 mL/g, *X*_4_ = 45% ethanol, *X*_5_ = 5 min) yielded predicted values of 105.36 mg GAE/g (TPC), 578.38 μmol AAE/g (FRAP), 565.01 μmol AAE/g (DPPH), and 14.64 mg/g (AAC), with an overall desirability of 0.736. Experimental validation under these conditions confirmed the model’s accuracy, with observed values of 87.79 ± 5.00 mg GAE/g (TPC), 741.4 ± 43.9 μmol AAE/g (FRAP), 616.1 ± 13.9 μmol AAE/g (DPPH), and 10.8 ± 0.7 mg/g (AAC) (Table 9). These results are remarkable, as a previous study [17] reported TPC values almost 4 times lower in AS. A similar result was drawn from Hefzalrahman et al. [38] who determined ~35 mg GAE/g dw on AP. In another study by Lyu et al. [39] determined ~78 mg GAE/g dw on AP. Moreover, Kamaraj et al. [40] 51.52 mg GAE/g on aqueous AP extracts. Ascorbic acid content was also determined on AP by another researcher, and the value was significantly lower than ours, at 4.1 ± 2.7 mg/100 g [41]. All of these reinforce the importance of this research, highlighting the positive impact of the optimized UAE model on AP.

**Figure 10:**
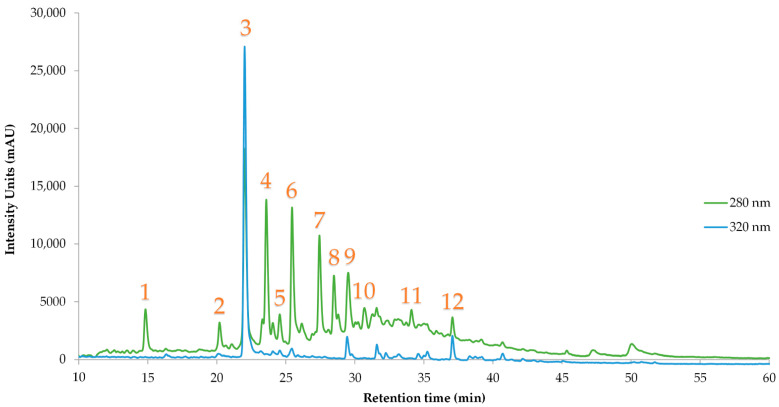
Representative HPLC chromatogram of the optimized avocado peel extract. Peaks correspond to identified polyphenolic compounds listed in Table 10. Retention times and peak identities were confirmed using authentic standards.

To further characterize the optimized extract, HPLC analysis was performed to identify and quantify individual polyphenolic compounds (Table 10, Figure 10). A total of 12 compounds were detected, yielding a cumulative concentration of 10.48 ± 0.47 mg/g dry weight. The most abundant constituents were catechin derivative 1 (2.21 ± 0.13 mg/g), chlorogenic acid (1.65 ± 0.06 mg/g), and catechin derivative 3 (1.60 ± 0.04 mg/g), followed by vanillic acid (1.20 ± 0.04 mg/g) and catechin derivative 2 (1.17 ± 0.06 mg/g). These findings highlight the dominance of flavan-3-ols and hydroxycinnamic acids in the extract. Classical antioxidants such as catechin (0.62 ± 0.02 mg/g), epicatechin (0.25 ± 0.01 mg/g), and 3-hydroxytyrosol (0.34 ± 0.02 mg/g) were also present, supporting the extract’s radical scavenging activity observed in DPPH and FRAP assays [42,43,44]. The detection of luteolin-7-glucoside (0.29 ± 0.01 mg/g) adds further bioactivity, while homovanillic acid (0.35 ± 0.03 mg/g) may reflect partial transformation or microbial activity during extraction [45,46]. Chlorogenic acid is abundant in AP, which was an anticipated outcome. Other scientists [47] determined chlorogenic acid and other (caffeoyl)quinic derivatives on AS. Vanillic acid is also another abundant polyphenol in the optimal extract. Jimenez et al. [21] reported this compound both as an AS and an AP, along with catechin and luteolin. Also, Trujillo-Mayol et al. [48] analyzed AP extracts with high-resolution mass spectrometry and reported several polyphenolic compounds, like catechin and some derivatives and chlorogenic acid. Overall, the polyphenolic profile is dominated by catechin-type structures, with five derivatives contributing significantly to the total. This suggests either structural diversity within the extract or partial polymerization/oxidation during processing. As illustrated in Figure A1, the overlay of UV–Vis spectra confirms the similarity in peak positions and absorbance profiles between the catechin standard and the identified compounds, justifying their designation as catechin-related structures. The high content of chlorogenic acid and vanillic acid further supports the extract’s potential for nutraceutical and functional food applications.

## 4. Conclusions

This study demonstrated the effective valorization of avocado peel and seed residues through optimized extraction and comprehensive characterization of their bioactive profiles. Multivariate modeling (PLS) guided the optimization of extraction conditions, identifying peel extracts as superior in total polyphenolic content (TPC), ferric reducing antioxidant power (FRAP), and ascorbic acid content (AAC), while seed extracts exhibited enhanced DPPH radical scavenging activity. Detailed HPLC analysis confirmed the presence of 12 distinct polyphenolic compounds in the peel extract, including chlorogenic acid, vanillic acid, catechin, epicatechin, 3-hydroxytyrosol, and luteolin-7-glucoside, with a total concentration of 10.48 ± 0.47 mg/g dry weight. The dominance of catechin-type structures and hydroxycinnamic acids supports the extract’s potent antioxidant activity and highlights its potential for nutraceutical applications. Fatty acid profiling revealed that seed oil was richer in monounsaturated fatty acids (MUFAs), particularly oleic acid, and exhibited greater oxidative stability, whereas peel oil contained significantly higher levels of polyunsaturated fatty acids (PUFAs), especially α-linolenic acid (ω-3), resulting in a favorable ω-6/ω-3 ratio and enhanced cardioprotective potential. Spectrophotometric analysis further highlighted the pigment richness of peel oil, with high concentrations of α-carotene (253.1 ± 15.95 mg/kg) and chlorophyll a (207.7 ± 9.76 mg/kg), while seed oil was dominated by lutein-type xanthophylls (29.2 ± 2.16 mg/kg). Despite the increased susceptibility of peel oil to oxidative degradation, its superior antioxidant and pigment profile suggests promising applications in functional foods, nutraceuticals, and natural colorant formulations. Seed oil, with its MUFA dominance and oxidative resilience, may serve as a stable lipid carrier or base oil in antioxidant-enriched systems. Overall, avocado peel and seed fractions offer complementary bioactive properties, and their targeted utilization could support sustainable by-product valorization strategies in the agri-food sector.

## Figures and Tables

**Table 1:** Coded levels of the independent variables used in the RSM design for UAE.

Independent Variables	Coded Units	Type	Coded Levels		
			−1	0	1
Waste	*X*_1_	Categorical	Peel	-	Seed
Ultrasonic power (*E*, %)	*X*_2_	Continuous	40	70	100
Liquid-to-solid ratio (*R*, mL/g)	*X*_3_	Continuous	10	40	70
Ethanol concentration (*C*, % *v/v*)	*X*_4_	Continuous	0	50	100
Extraction time (*t*, min)	*X*_5_	Continuous	5	15	25

**Table 2:** Fatty acid composition of avocado peel and seed oils (%total fatty acids, mean ± SD, *n* = 3). Significant differences (*p* < 0.05) are marked with (*).

Fatty Acid	Peel (%)	Seed (%)
C10:0 (Decanoic)	n.d.	0.11 ± 0.00 *
C12:0 (Lauric)	3.17 ± 0.19 *	0.17 ± 0.01
C14:0 (Myristic)	0.34 ± 0.01 *	0.07 ± 0.00
C16:0 (Palmitic)	19.38 ± 0.79	20.73 ± 0.48
C18:0 (Stearic)	0.96 ± 0.04	4.70 ± 0.15 *
C20:0 (Arachidic)	n.d.	0.05 ± 0.00 *
C22:0 (Behenic)	0.23 ± 0.01 *	0.03 ± 0.00
C24:0 (Lignoceric)	0.20 ± 0.01	0.94 ± 0.04 *
∑ SFA	24.28 ± 1.06	26.80 ± 0.69 *
C16:1 (Palmitoleic)	1.68 ± 0.05 *	n.d.
C18:1 (Oleic)	42.05 ± 3.11	48.09 ± 2.31
C22:1 (Erucic)	0.05 ± 0.00 *	n.d.
∑ MUFA	43.78 ± 3.16	48.09 ± 2.31
C18:2 (Linoleic, ω-6)	21.88 ± 1.53	24.04 ± 0.87
C18:3 (α-Linolenic, ω-3)	9.72 ± 0.70 *	0.61 ± 0.02
∑ PUFA	31.60 ± 2.23 *	24.65 ± 0.88
∑ UFA	75.38 ± 5.40	72.74 ± 3.19
PUFA:SFA	1.30 ± 0.04 *	0.92 ± 0.01
MUFA:PUFA	1.39 ± 0.00	1.95 ± 0.02 *
ω-6/ω-3	2.25 ± 0.00 *	39.43 ± 1.54 *

n.d. for not detected.

**Table 3:** Oxidative stability and antioxidant activity of avocado peel and seed oils (mean ± SD, *n* = 3). Significant differences (*p* < 0.05) are marked with (*).

Parameter	Peel	Seed
DPPH (mmol TEAC/kg oil)	15.49 ± 0.91	14.97 ± 0.66
PV (mmol H_2_O_2_/kg oil)	16.95 ± 0.76 *	13.50 ± 0.27
CD (mmol/kg oil)	33.56 ± 2.52 *	11.51 ± 0.51
CT (mmol/kg oil)	10.52 ± 0.36 *	1.67 ± 0.13

**Table 4:** Spectral characteristics and estimated concentrations of pigments in avocado peel and seed oils. Carotenoid identity was inferred from absorption maxima and fine-structure ratios (%III/II), with lutein assigned to seed oil and α-carotene to peel oil. Chlorophyll a was quantified in peel oil using Wellburn and Lichtenthaler’s equations [28]. Values represent mean ± standard deviation (*n* = 3), expressed in mg pigment per kg oil.

Sample	Assigned Pigment	λ_max_ (nm)	%III/II	Concentration (mg/kg Oil)
Seed oil	Lutein	420, 442, 470	83.80%	29.2 ± 2.16
Peel oil	α-Carotene	432, 470	n.d.	253.1 ± 15.95
Peel oil	Chlorophyll a	661	—	209.4 ± 9.76

n.d. for not detected.

**Table 5:** Experimental design matrix (coded and actual values) and responses (TPC, FRAP, DPPH, AAC) for ultrasound-assisted extraction of avocado peel and seed residues.

Design Point	Independent Variables					Actual Responses *			
	Waste (*X*_1_)	Power (*X*_2_, %)	Ratio (*X*_3_, mL/g)	Ethanol (*X*_4_, % *v/v*)	Time (*X*_5_, min)	TPC	FRAP	DPPH	AAC
1	Seed	70	40	50	15	53.84	275.03	235.1	6.18
2	Seed	40	70	0	5	17.18	407.16	54.21	5.82
3	Peel	40	10	100	15	3.72	77.69	160.59	1.9
4	Seed	100	10	100	25	32.01	369.81	291.93	2.72
5	Peel	40	40	0	15	43.83	546.7	389.09	12.73
6	Peel	40	70	50	5	71.87	568.65	586	14.81
7	Seed	70	40	50	15	53.79	272.46	243.16	6.43
8	Peel	70	70	100	15	10.2	130.45	488.92	11.36
9	Peel	100	40	100	25	18.17	203.12	401.18	8.08
10	Seed	100	70	100	5	41.05	408.65	1103.76	8.09
11	Peel	70	70	0	25	51.26	449.8	480.74	17.19
12	Peel	40	10	0	5	58.8	68.32	327.66	5.59
13	Peel	40	40	50	25	88.4	529.99	422.69	10.68
14	Seed	100	10	0	5	26.22	95.02	96.69	2.45
15	Seed	70	40	50	15	51.18	257.28	230.27	6.02
16	Seed	40	10	0	25	25.78	149.16	105.78	2.94
17	Peel	100	70	50	15	103.14	566.51	420.87	13.78
18	Seed	100	70	0	25	28.89	174.59	136.52	4.67
19	Peel	100	10	100	5	11.53	93.69	53.58	2.02
20	Peel	100	10	0	25	67.32	371.43	278.81	5.33
21	Seed	40	70	100	25	51.04	265.98	240.95	7.95
22	Seed	70	40	50	15	52.65	263.33	282.13	6.16
23	Peel	100	70	0	5	78.97	514.63	423.45	15.59
24	Seed	40	10	100	5	36.57	67.29	102.85	2.48
25	Peel	70	10	50	25	90.86	602.39	207.89	4.87
26	Peel	70	40	100	5	17.41	61.61	404	5.54

* Values represent the mean of triplicate determinations; TPC, total polyphenol content in mg GAE/g dw; FRAP, ferric reducing antioxidant power in µmol AAE/g dw; DPPH antiradical activity in µmol AAE/g dw; AAC, ascorbic acid content in mg/g dw.

**Table 6:** ANOVA summary (*p*-values) for the fitted quadratic models of TPC, FRAP, DPPH, and AAC.

Term/Source	TPC	FRAP	DPPH	AAC
Model (overall)	<0.0001 ***	<0.0001 ***	<0.0001 ***	<0.0001 ***
*X*_1_ − Waste	0.0008 **	0.0085 **	0.0042 **	<0.0001 ***
*X*_2_ − Power	0.0973 (ns)	0.828 (ns)	0.0077 **	0.154 (ns)
*X*_3_ − Ratio	0.1962 (ns)	0.0011 **	<0.0001 ***	<0.0001 ***
*X*_4_ − Ethanol concentration	0.0041 **	0.0268 *	0.0003 ***	0.0001 ***
*X*_5_ − Time	0.4163 (ns)	0.067 (trend)	0.047 *	0.176 (ns)
*X*_1_ × *X*_2_	0.0733 (trend)	–	0.0002 ***	–
*X*_1_ × *X*_3_	–	–	0.222 (ns)	<0.0001 ***
*X*_1_ × *X*_4_	<0.0001 ***	0.0007 ***	0.0002 ***	<0.0001 ***
*X*_1_ × *X*_5_	–	0.053 (trend)	0.025 *	0.083 (trend)
*X*_2_ × *X*_3_	0.053 (trend)	0.067 (trend)	0.012 *	–
*X*_2_ × *X*_4_	0.0838 (trend)	0.233 (ns)	0.018 *	0.046 *
*X*_2_ × *X*_5_	–	0.062 (trend)	–	0.011 *
*X*_3_ × *X*_4_	0.0693 (trend)	0.0227 *	0.0002 ***	–
*X*_3_ × *X*_5_	–	0.0018 **	0.0045 **	–
*X*_4_ × *X*_5_	–	–	0.027 *	–
*X*_2_^2^	0.0315 *	0.0333 *	–	–
*X*_3_^2^	–	–	0.280 (ns)	0.106 (trend)
*X*_4_^2^	<0.0001 ***	0.0008 ***	–	0.039 *
*X*_5_^2^	0.0087 **	–	0.048 *	–
Lack of fit	0.0043 *	0.0015 *	0.048 *	0.0177 *
R^2^/Adj. R^2^	0.961/0.919	0.934/0.850	0.972/0.924	0.989/0.979

Significance codes: *** *p* < 0.001; ** *p* < 0.01; * *p* < 0.05; (trend) 0.05 < *p* < 0.1; ns = not significant.

**Table 7:** Optimum extraction conditions and maximum predicted responses for avocado peel and seed extracts.

Response	Waste (*X*_1_)	Power (*X*_2_, %)	Ratio (*X*_3_, mL/g)	Ethanol (*X*_4_, % *v*/*v*)	Time (*X*_5_, min)	Desirability	Maximum Predicted Response
TPC	Peel	100	70	39	5	0.9465	105.98 ± 12.94
FRAP	Peel	40	70	20	24	0.9458	673.89 ± 141
DPPH	Seed	100	70	100	5	0.8776	1071.31 ± 131.16
AAC	Peel	40	70	0	25	0.9919	17.9 ± 1.18

TPC, total polyphenol content in mg GAE/g dw; FRAP, ferric reducing antioxidant power in µmol AAE/g dw; DPPH antiradical activity in µmol AAE/g dw; AAC, ascorbic acid content in mg/g dw.

**Table 8:** Correlation matrix among antioxidant assays of avocado peel and seed extracts.

Responses	TPC	FRAP	DPPH	AAC
TPC	–	0.738	0.308	0.530
FRAP		–	0.425	0.688
DPPH			–	0.587
AAC				–

**Table 9:** Optimized extraction conditions, predicted responses from the PLS regression model, overall desirability, and experimental validation values for avocado peel extracts.

Response	Waste (*X*_1_)	Power (*X*_2_, %)	Ratio (*X*_3_, mL/g)	Ethanol (*X*_4_, % *v*/*v*)	Time (*X*_5_, min)	PLS Regression	Desirability	Experimental Values
TPC	Peel	100	70	45	5	105.36	0.7359	87.79 ± 5
FRAP						578.38		741.4 ± 43.93
DPPH						565.01		616.07 ± 13.94
AAC						14.64		10.8 ± 0.66

TPC, total polyphenol content in mg GAE/g dw; FRAP, ferric reducing antioxidant power in µmol AAE/g dw; DPPH antiradical activity in µmol AAE/g dw; AAC, ascorbic acid content in mg/g dw.

**Table 10:** Polyphenolic composition of avocado peel extract under optimized conditions, as determined by HPLC (mean ± SD, *n* = 3).

No	Polyphenolic Compound	mg/L Extract	mg/g Dry Weight
1	3-Hydroxytyrosol	8.46 ± 0.61	0.34 ± 0.02
2	Catechin	15.54 ± 0.5	0.62 ± 0.02
3	Chlorogenic acid	41.48 ± 1.62	1.65 ± 0.06
4	Vanillic acid	30.05 ± 1.11	1.2 ± 0.04
5	Homovanillic acid	8.88 ± 0.64	0.35 ± 0.03
6	Epicatechin	6.18 ± 0.16	0.25 ± 0.01
7	Catechin derivative 1	55.34 ± 3.27	2.21 ± 0.13
8	Catechin derivative 2	29.38 ± 1.62	1.17 ± 0.06
9	Catechin derivative 3	40.18 ± 1	1.6 ± 0.04
10	Catechin derivative 4	12.42 ± 0.32	0.5 ± 0.01
11	Catechin derivative 5	7.76 ± 0.51	0.31 ± 0.02
12	Luteolin-7-glucoside	7.34 ± 0.35	0.29 ± 0.01
	Total identified	263.03 ± 11.71	10.48 ± 0.47

## Data Availability

The original contributions presented in this study are included in the article. Further inquiries can be directed to the corresponding author.

## Abbreviations

The following abbreviations are used in this manuscript:

AAC	Ascorbic Acid Content
AAE	Ascorbic Acid Equivalents
ANOVA	Analysis of Variance
AP	Avocado Peel
AS	Avocado Seed
CD	Conjugated Dienes
CT	Conjugated Trienes
DAD	Diode Array Detector
DPPH	2,2-Diphenyl-1-picrylhydrazyl
dw	Dry Weight
FAME	Fatty Acid Methyl Esters
FRAP	Ferric Reducing Antioxidant Power
GAE	Gallic Acid Equivalents
GC-FID	Gas Chromatography with Flame Ionization Detector
HPLC	High-Performance Liquid Chromatography
LOD	Limit of Detection
LOQ	Limit of Quantification
MUFA	Monounsaturated Fatty Acids
PCA	Principal Component Analysis
PLS	Partial Least Squares
PUFA	Polyunsaturated Fatty Acids
PV	Peroxide Value
ROS	Reactive Oxygen Species
RSM	Response Surface Methodology
SD	Standard Deviation
SFA	Saturated Fatty Acids
TE	Trolox Equivalents
TPC	Total Polyphenolic Content
TPTZ	2,4,6-Tris(2-pyridyl)-s-triazine
UAE	Ultrasound-Assisted Extraction
UV-Vis	Ultraviolet-Visible Spectroscopy
VIP	Variable Importance in Projection

## Appendix A

**Table A1:** Calibration equations, correlation coefficients (R^2^), retention times (RT), UV absorbance maxima (UV_max_), limits of detection (LOD), and quantification (LOQ) for all identified polyphenolic compounds. For catechin derivatives 1–5, identification was based on spectral similarity to the catechin standard, as shown in Figure A1.

Polyphenolic Compound	Equation (Linear)	R^2^	RT (min)	UV_max_ (nm)	LOD (mg/L)	LOQ (mg/L)
3-Hydroxytyrosol	y = 183,448.37x – 251,422.04	0.997	14.552	278	2.87	8.69
Catechin	y = 11,920.78x − 128.19	0.997	20.933	278	2.54	7.71
Chlorogenic acid	y = 50,320.40x − 23,038.36	0.994	21.947	325	3.67	11.11
Vanillic acid	y = 28,178.39x + 15,571.90	0.999	23.900	270	2.31	6.99
Homovanillic acid	y = 18,843.08x + 6856.98	0.999	24.458	279	1.18	3.59
Epicatechin	y = 142,099.00x + 4705.94	0.999	25.821	278	0.19	0.58
Luteolin-7-glucoside	y = 34,875.94x − 16,827.36	0.999	36.949	347	1.28	3.89
